# A tailored programme to implement recommendations for multimorbid patients with polypharmacy in primary care practices—process evaluation of a cluster randomized trial

**DOI:** 10.1186/s13012-017-0559-y

**Published:** 2017-03-06

**Authors:** Cornelia Jäger, Jost Steinhäuser, Tobias Freund, Sarah Kuse, Joachim Szecsenyi, Michel Wensing

**Affiliations:** 1 Department of General Practice and Health Services Research, University Hospital Heidelberg, Im Neuenheimer Feld 130.3, Turm West, 4. OG, 69120 Heidelberg, Germany; 2 University Hospital Schleswig-Holstein/Campus Lübeck, Institute of Family Medicine, Ratzeburger Allee 160, Haus 50, 23538 Lübeck, Germany

**Keywords:** Process evaluation, Polypharmacy, Implementation, Brown bag review, Medication list, Communication, Primary care

## Abstract

**Background:**

We developed and evaluated a tailored programme to implement three evidence-based recommendations for multimorbid patients with polypharmacy into primary care practices: structured medication counselling including brown bag reviews, the use of medication lists and medication reviews. No effect on the primary outcome was found. This process evaluation aimed to identify factors associated with outcomes by exploring nine hypotheses specified in the logic model of the tailored programme.

**Methods:**

The tailored programme was developed with respect to identified determinants of practice and consisted of a workshop for practice teams, elaboration of implementation action plans, aids for medication reviews, a multilingual info-tool for patients on a tablet PC, posters and brown paper bags as reminders for patients. The tailored programme was evaluated in a cluster randomized trial. The process evaluation was based on various data sources: interviews with general practitioners and medical assistants of the intervention group and a survey with general practitioners of the intervention and control group, written reports on the implementation action plans, documentation forms for structured medication counselling and the log file of the info-tool.

**Results:**

We analyzed 12 interviews, 21 questionnaires, 120 documentation forms for medication counselling, 5 implementation action plans and one log file of the info-tool. The most frequently reported effect of the tailored programme was the increase of awareness for the health problem and the recommendations, while implementation of routine processes was only reported for structured medication counselling. The survey largely confirmed the usefulness of the applied strategies, yet the interviews provided a more detailed understanding of the actual use of the strategies and several suggestions for modifications of the tailored programme.

**Conclusions:**

The tailored programme seemed to have induced awareness as a first step of behaviour change. Several modifications of the tailored programme may enhance its effectiveness such as conducting outreach visits instead of a workshop, improved targeting, provision of evidence, integration of tools into the practice software and information materials in tailored formats.

**Trial registration:**

This study is linked to an outcome evaluation study with the registration ISRCTN34664024, assigned 14/08/2013.

**Electronic supplementary material:**

The online version of this article (doi:10.1186/s13012-017-0559-y) contains supplementary material, which is available to authorized users.

## Background

### Current care and best practice

Patients with multiple chronic conditions and polypharmacy are at high risk for preventable adverse drug reactions (ADR) [1], potentially avoidable hospital admissions and preventable deaths [2, 3]. The risk factors and causes leading to such undesired events are well described [4–6] and frequently involve the challenge of coordinating the care of this patient group among different health care providers and prescribers. Thus, general practitioners (GPs), who act as coordinators and central medical care providers in the health system, play an important role in the care of multimorbid patients with polypharmacy, especially in Germany, a country without formal gate-keeping system and free access to specialist care [7].

Therefore, it is important to optimize medication-related processes in general practices when trying to improve the care of these patients [8]. We identified three recommendations from the literature, which were later on also included into a German guideline on multimedication in elderly primary care patients [9]. We developed and evaluated a tailored programme (TP) to improve the implementation of recommendations, which address different areas of care:

- *Recommendation A on communication about medication*: All patients with polypharmacy and additional risk factors for medication problems should receive *structured medication counselling* (*SMC*) at least once per year. Beside medication-related information, SMC comprises a complete inventory of the medication actually taken by the patient (so called “brown bag review”) and an assessment of adherence and possible application problems. A separate appointment should be planned for SMC [9, 10].
- *Recommendation B on documentation of medication*: All patients with polypharmacy should take along an updated, complete and comprehensible *medication list*, concordant with the template of the Drug Commission of the German Medical Association [11].
- *Recommendation C on prescribing*: Physicians should perform structured *medication reviews* with the aid of tools, such as the PRISCUS list [12] or the Medication appropriateness index (MAI) [13], to reduce potentially inappropriate medication regimes. PRISCUS lists 83 substances which should be avoided in older, multimorbid patients. The MAI is a compilation of implicit criteria which should be taken into account when reviewing a medication regimen.

### Tailoring

Interventions to improve the appropriate use of polypharmacy frequently do not show clinically significant effects and are criticized for the lack of guidance on intervention development and reporting [14, 15]. “Tailoring” is an approach to increase the effectiveness of interventions by systematic identification of barriers and enablers of practice (also referred to as “determinants of practice”) and careful selection of strategies to address these determinants [16].

The term tailoring has to be distinguished from “modification” and “adaptions” of interventions. While tailoring refers to the development of the interventions in the design phase, “modifications can include adaptations, which are planned or purposeful changes to the design or delivery of an intervention, but they can also include unintentional deviations from the interventions as originally designed. That is, some modifications occur with the intention to retain fidelity to the fundamental elements or spirit of the intervention, whereas others may be unplanned changes made in reaction to a specific circumstance” [17].

**Fig. 1:**
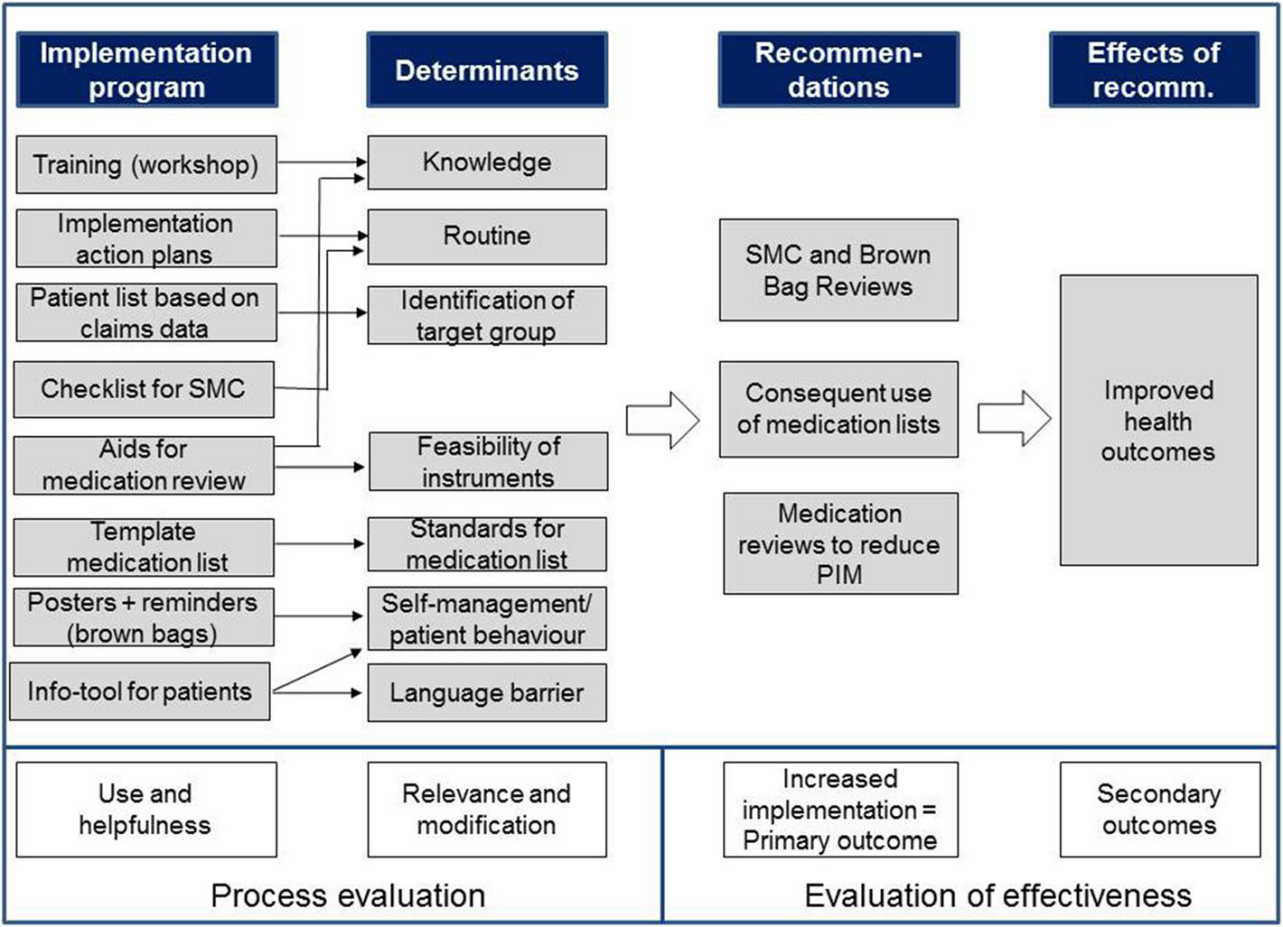
Logic model of the tailored programme. The figure describes the assumed mechanism of the intervention and the structure of the evaluation: An implementation programme consisting of various strategies to address specific determinants of practice will increase the implementation of evidence-based recommendations. Based on previous evidence, it can be assumed that increased implementation will result in improve health outcomes. Therefore, the primary outcome is the “degree of implementation” while the effects of the recommendations are secondary outcomes. The relevance and modification of determinants and the use and helpfulness of the strategies are subject of the process evaluation.

This study is part of the “Tailoring Interventions for Chronic Diseases” (TICD) project [18], during which five TPs for implementation of recommended practices have been developed and evaluated in randomized controlled trials [19–23]. The process of tailoring used in TICD has been described in detail elsewhere [24–26] and comprised the systematic identification of determinants and strategies involving qualitative and quantitative methods and various target groups. In our study, GPs, specialists, nurses and researchers were targeted to identify determinants and strategies. The final selection of determinants and strategies was done by the researchers. A “logic model” which illustrates the assumed mechanisms of the TP and the assumed relation between the selected strategies, the determinants intended to be modified, the recommendations intended to implement and the effect on health outcomes was elaborated for each TP (Fig. 1).

### Strategies to implement recommendations on polypharmacy

The TP of our trial consisted of the following strategies [22]: The intervention period started with a four-hour workshop for practice teams, delivered by two of the authors (CJ, JS). It comprised an analysis of barriers and solutions for the implementation of the recommendations using a card technique, role plays and case examples to convey pharmacological skills and knowledge as well as organizational information related to the study. Details about the workshop have been published separately [27]. At the end of the workshop, the participants received information materials for patients (posters, brown bags and a multilingual info-tool on a tablet PC). After the workshop, the participants were asked to organize a team meeting to elaborate “implementation action plans”, i.e. an individual concept of how they were going to implement the recommendations into their practice. They were asked to send a written report about their decisions to the study centre. Furthermore, the intervention and control group was provided with a list of patients meeting the inclusion criteria derived from insurance claim data.

### Effectiveness of the tailored programme

Despite the comprehensive identification of determinants and strategies prior to designing the TP, we did not observe an effect of the TP on the primary outcome, which was a combination of ten indicators reflecting the degree of implementation. Only two indicators measuring the implementation of SCM increased significantly in the intervention group, and the item “I show my medication list regularly when buying a drug in the pharmacy” of a questionnaire measuring patients’ self-reported use of the medication list improved significantly in the intervention group. Yet due to limitations of the trial (e.g. sample size not reached), results have to be interpreted carefully [28].

The process evaluation presented in this article explores the following nine hypotheses—which had been specified in the logic model during the design phase of the intervention (Fig. 1)—in order to explain the low effectiveness and to provide suggestions for modification of the programme:

1. Training increased expert knowledge and routines
2. Implementation action plans increased routine
3. Patient lists helped to identify suitable patients for SMC
4. The checklist helped to establish routines for SMC in the practice
5. The aids for medication reviews increased expert knowledge and feasibility of instruments for systematic medication reviews
6. The template helped to bring medication lists in line with the standards
7. The information material for patients increased the self-management abilities of patients and reduced language barriers and difficulties of comprehension
8. Implementation of the recommendations has improved
9. Implementation of the recommendations leads to improved health outcomes

## Methods

### Study design

The outcomes of the TP have been evaluated in a cluster randomized controlled trial with a follow-up time of 9 months and randomization on the level of quality circles (registration number ISRCTN 34664024). The design and evaluation of primary and secondary outcomes have been described separately [28]. This article focuses on the process evaluation of the trial for which a mixed method design based on interviews and a survey with the participating health care professionals was used. While all research questions of the survey were also part of the interviews in order to compare the responses, the interviews covered additional aspects (e.g. the question about alternative strategies) not asked in the survey. It followed a previously published study protocol which guided the TICD trials [26].

### Sample

The TP was delivered to primary care practices in one larger area in South Germany that participated in a special care contract of one large German health insurance (HZV AOK Baden-Württemberg) [29] and in “quality circles” comprising small group meetings of GPs of one local area [30]. The inclusion criteria for patients were age >50 years, diagnosis of at least three chronic conditions based on a previously published diagnosis list [31], repeated prescription of four different drugs, high risk for ADR according to the subjective assessment of the treating GP and mental and physical ability to participate in the study. The target group for this study were GPs for interviews and the survey and MA for interviews.

### Data collection

The intervention period started in the end of January 2014 with the workshop and ended in October 2014 with the closure of the data bank for medication counselling.

We conducted a *survey* with GPs of the intervention and control group which was completed in the practices on the provided tablet PC and delivered to a secure server of the University of Heidelberg within 2 months after the intervention period had ended. It focused on the relevance and modification of determinants and the usefulness and modification of strategies. Parts of the survey were applied to the intervention and control group and parts to the intervention group only (Table 1).

**Table 1:** Survey on determinants and strategies

Item number	Survey item	Group	Agree % (*n*)	Partly agree % (*n*)	Do not agree % (*n*)
1 (a)	I need more knowledge to implement these recommendations into my practice	IG + CG	14.3 (3)	57.1 (12)	28.6 (6)
		IG	20.0 (2)	60.0 (6)	20.0 (2)
		CG	9.1 (1)	54.5 (6)	36.4 (4)
1 (b)	The workshop of the PomP study conveyed useful knowledge for the implementation of the recommendations into my practice	IG	60.0 (6)	40.0 (4)	0.0 (0)
2 (a)	The lack of work routines hinders the implementation of the recommendations into my practice	IG + CG	19.0 (4)	33.3 (7)	47.6 (10)
		IG	10 (1)	20.0 (2)	70 (7)
		CG	27.3 (3)	45.5 (5)	27.3 (3)
2 (b)	The elaboration of implementation action plans helped to establish routines for the implementation of the recommendations	IG	30.0 (3)	60.0 (6)	10.0 (1)
3 (a)	It is difficult for me to select the patients who profit most from SMC	IG + CG	0.0 (0)	23.8 (5)	76.2 (16)
		IG	0.0 (0)	40.0 (4)	60.0 (6)
		CG	0.0 (0)	9.1 (1)	90.9 (10)
3 (b)	The patient list provided at the beginning of the study helped me to identify suitable patients for SMC	IG	40.0 (4)	40.0 (4)	20.0 (2)
4 (a)	Instruments for medication reviews such as the PRISCUS list or the MAI are not feasible enough to use them regularly	IC + CG	42.9 (9)	47.6 (10)	9.5 (2)
		IG	30.0 (3)	60.0 (6)	10.0 (1)
		CG	54.5 (6)	36.4 (4)	9.1 (1)
4 (b)	The online-tools and checklists provided on the tablet PC helped me to conduct medication reviews.	IG	50.0 (5)	30.0 (3)	20.0 (2)
5 (a)	The lacking standardization of medication lists impede the implementation of recommendation B into my practice	IG + CG	23.8 (5)	38.1 (8)	38.1 (8)
		IG	20.0 (2)	40.0 (4)	40.0 (4)
		CG	27.3 (3)	36.4 (4)	36.4 (4)
5 (b)	The provided template for medication lists helped me to bring the medication list of my practice in line with defined minimum standards.	IG	50.0 (5)	20.0 (2)	30.0 (3)
6 (a)	The lacking self-management abilities of patients impede the implementation of the recommendations into my practice	IG + CG	19.0 (4)	71.4 (15)	9.5 (2)
		IG	20.0 (2)	70.0 (7)	10.0 (1)
		CG	18.2 (2)	72.7 (8)	9.1 (1)
6 (b)	The information material (posters, tablet pc) induced patients to take their medication list with them.	IG	50.0 (5)	30.0 (3)	20.0 (2)
7 (a)	Language barriers of non-German-speaking patients impede the implementation of recommendation A into my practice	IG + CG	52.4 (11)	23.8 (5)	23.8 (5)
		IG	50.0 (5)	20.0 (2)	30.0 (3)
		CG	54.5 (6)	27.3 (3)	18.2 (2)
7 (b)	The info-tool on the tablet PC helped to reduce problems due to language barriers	IG	30.0 (3)	40.0 (4)	20.0 (2)
8 (a)	Difficulties of comprehension between me and my patients impede the implementation of recommendation A into my practice	IG + CG	28.6 (6)	28.6 (6)	42.9 (9)
		IG	30.0 (3)	30.0 (3)	40.0 (4)
		CG	27.3 (3)	37.3 (3)	45.5 (5)
8 (b)	The info-tool on the tablet PC facilitated the communication with my patients	IG	40.0 (4)	50.0 (5)	10.0 (1)

The “(a) items” were applied to the intervention and control group; the “(b) items” were applied to the intervention group only

*IG* intervention group, *CG* control group

Furthermore, we conducted individual *interviews* with GPs and medical assistants (MA) of the intervention group shortly before the intervention period ended from end of August to end of September 2014. In group practices, only one GP and one MA were interviewed to limit the effort for each practice. The decision which GP/MA would do the interview was taken by the participants. Interviews were done in the practices by a researcher not directly involved in the delivery of the TP. They followed a semi-structured interview guide focusing on the following key questions:

- What is the participants’ view of the recommendations on polypharmacy?
- How did the participants use the strategies offered to them?
- How did the participants evaluate the feasibility and helpfulness of the applied strategies?
- Did the strategies from their perspective help to modify the determinants as intended?
- How successful was the implementation of the recommendations from the perspective of the participants?

Furthermore, we asked the practice teams to send us a written report about the *implementation action plan* they had elaborated latest 2 weeks after the workshop. We asked all GPs to *document each SMC* they conducted via an online form on the tablet PC. Additionally, we analyzed the *log file* of the tablet PC to assess how often the info-tool was used.

### Data analysis

Interviews were audiotaped, transcribed and analyzed independently by two researchers according to the principles of qualitative content analysis [32]. The research questions described above served as deductive framework to which sub-categories were added inductively by two researchers. The coding was discussed among the researchers and the classification system was adapted continuously. After the analysis of the last interview, a final version of the classification system was developed and the assignment of the quotations was checked for consistency. The survey, the forms for the documentation of SMC and the log file were analyzed descriptively. The implementation action plans were summarized.

## Results

### Participants

We enrolled 22 GPs from 18 practices and 344 patients into the study. In the intervention group, 143 patients and 10 GPs from 6 practices and in the control croup, 130 patients and 11 GPs from 11 practices completed the study. Patients were on average 72 (52-94) years old, diagnosed with 5.7 chronic diseases and prescribed 7.3 drugs. GPs were on average of 55 (44–68) years old and by the majority male (82%, *n* = 18). The fact that several GPs of the intervention group worked in group practices and all GPs of the control group in single practices is a result of the randomization on quality circle level and a limitation of the study. In total, 12 interviews were conducted which lasted on average 32 min (16–58). All participating GPs (*n* = 21) completed the survey. We received written reports on the implementation action plans from five out of six practices. In total, 120 forms for SMC were completed by GPs of the intervention group.

### Hypotheses of the logic model

The logic model of the TP (Fig. 1) specifies nine main hypotheses which we will deal with consecutively combining the findings from the different data sources for the purpose of triangulation.

#### Hypothesis 1: training increased expert knowledge and routines

The aim of the workshop offered to the practice teams was to convey the necessary knowledge about the recommendations, to discuss possible barriers for their implementation and to develop a menu of strategies the participants could use to avoid errors and to overcome barriers and thus to establish work routines for the recommendations in their practice. Furthermore, role plays to practice “brown bag reviews” and case examples to practice structured medication reviews were done. Directly after the workshop, the participants had been asked to complete an evaluation form on course and content of the workshop. This evaluation was almost exclusively positive [27]. In the survey conducted 9 months after the workshop, 71% of the participants stated that they considered the lack of knowledge at least partly a relevant determinant and that the workshop helped to address this determinant (Table 1, item 1). In the interviews conducted 9 months after the workshop, the evaluation was more heterogeneous. Some participants hardly remembered the content of the workshop. While the MAs especially appreciated the group work and the materials, GPs tended to complain about the efforts the participation in the workshop caused. Yet, they appreciated the exchange with colleagues and suggested to integrate the training into the small group meetings of the quality circles.

What was most helpful for me: the exchange with the other colleagues (GP)

I would like to reinforce this in our quality circle and discuss specific or difficult patients (GP)

#### Hypothesis 2: implementation action plans increased routine

After the workshop, the practice teams were asked to organize a team meeting and to elaborate an implementation action plan. For this purpose, they received a written compilation of the barriers and solutions collected during the workshop which has been published before [27]. We asked them to send us a written report with the following key aspects: (a) the barriers/error sources that were most important in their specific practice, (b) the activities they planned to overcome the barriers, (c) the responsible person for each activity and (d) the time schedule for each activity. The aim of this strategy was to raise awareness for the care deficiencies and error sources in their own practice among all practice staff and to establish work routines for the recommendations.

In the survey, nearly half of the GPs stated that the lack of work routines was not a relevant barrier in their practice. However, 90% of the GPs of the intervention group found that the elaboration of implementation action plan helped at least partly to establish such work routines (see Table 1, item 2). In the interviews, the participants expressed different views on this strategy. Some GPs appreciated that the implementation action plan helped them to raise awareness and to motivate the practice staff for change.

This helped us a lot. The motivation of the staff was stronger and as you can see we have realised most of the issues we have elaborated. That was most helpful (GP).

The essential thing is to become aware of the difficulties (…) this is for sure what [helped] most (GP).

On the other hand, it became clear that not all practices had elaborated their concept during a team meeting, but that in some cases GPs had assigned the tasks without asking the agreement of the MA. Some expressed difficulties to identify room for improvement in their practice.

All this happens already automatically in our practice and this is why there was nothing to change (MA)

Looking at the written reports, there was substantial variance concerning the length, the level of detail and the readability of the specifications. The following barriers/error sources were mentioned: writing prescriptions, repeated prescriptions, double prescriptions, dosing errors, complete medication not known, communication problems, patients do not carry their medication list with them, frequent change of trade names of the medications and software errors. The activities they decided to undertake frequently referred to the process of issuing prescriptions, such as to write repeated prescriptions only for medications specified on the medication list and that the MA should ask patients for their medication list when printing prescriptions, to inform patients that the issuing of prescriptions will take 1 day and to create a flyer to encourage them to use the practice hotline for ordering prescriptions. Other activities they specified were as follows: create a medication list including prescriptions of other doctors, MA reminds GP of medication lists, ask pharmacists to note down the trade name on the medication list when issuing the medication, document the distribution of sample packages, note down allergies and the reason for prescription on the medication list, combine brown bag reviews with check-ups, draft a separate form for insulin injections, update medication lists of nursing home patients by phone every 3 months or during each home visit, install links to online tools on all computers in the practice and monitor ADR.

#### Hypothesis 3: patient lists helped to identify suitable patients for SMC

We provided all practices (also the control practices) with a list of patients meeting the inclusion criteria based on insurance claim data to support them to identify suitable patients for SMC. According to the survey, for the majority of GPs, the identification of these patients was not a relevant determinant (Table 1, item 3). Yet, 80% of the GPs from the intervention group regarded the patient list as (partly) helpful. In the interviews, however, the identification of the target group was a frequently mentioned issue. Some physician said they would have preferred to include other patients than those on the list.

I can just pick patients here and then, when I have the feeling, I should have a look here (…) But this patient with the insulin, that was actually no risk patient for me, he would not have struck me due to the lab results. So the question is: How to reach these people? That’s virtually impossible! (GP)

There were not so many news or changes in those specific patients (…), but you are more sensitized and you pay more attention in other patients as well (GP)

#### Hypothesis 4: the checklist helped to establish routines for SMC in the practice

In the interviews, most GPs expressed a negative view of the checklist for SMC (provided as Additional file 1), although they were rather talking about checklists in general. Many GPs stated that there were too many checklists for too many issues which were not feasible and too time consuming for use in practice. Furthermore, many GPs felt that providing them with checklists was a way of questioning their experience and competence.

I think you shouldn’t try to take away someone’s experience because of some checklist or guideline (GP).

I need a checklist only if I don’t have knowledge (…) you should trust the doctors more, they have knowledge and that they don’t need checklists! (GP)

Another issue was that they feared the use of a checklist aiming at standardizing or structuring the conversation would impede the individual care for the patient. Some GPs mentioned that checklists might be useful for young, unexperienced doctors. MAs were more positive towards the checklist and stated that they would have used it, if it would have been digital and available via the computers in the practice.

#### Hypothesis 5: the aids for medication reviews increased expert knowledge and feasibility of instruments for systematic medication reviews

During the workshop, we introduced some aids (a sheet with a modified MAI and the PRISCUS drugs in alphabetic order and online resources) to the GPs which they could use for a systematic medication review (see Additional file 2). In the survey, the vast majority of GPs stated that such tools were generally not feasible enough to use them regularly but the majority of the GPs of the intervention group considered the provided materials helpful for medication reviews (Table 1, item 4). In the interviews, the main barrier for using the tools was that they were not integrated into the practice software. One GP stated to have added the links to the online resources to the favourites bar of each computer in the practice.

No, we didn’t use the tablet very much. If I go online, I do it with [my computer] (…) so we didn’t use it (…) because [these tools] were disconnected from our system (GP).

#### Hypothesis 6: the template helped to bring medication lists in line with the standards

Medication lists frequently lack important information. We provided the practice teams with a template defining the minimum standards for a comprehensive medication list (e.g. that the list should contain the name of the active ingredient and not only trade names and the reason for drug use) and asked them to bring the medication lists they are using in line with those standards. In the survey (Table 1, item 5), about 60% of the GPs considered the lack of standards for medication lists a relevant barrier and 70% stated that the template was helpful to adjust the medication lists used in their practices. In the interviews, many respondents reported to add the reason for prescription more often now.

The analysis of the primary outcome, however, showed that GPs rarely made any changes concerning the layout and content of their medication lists [28]. In the interviews and in the implementation action plans, it became evident that two major barriers for bringing the medication lists in line with the template had not been addressed. The first one was the functions of the practice software which frequently did not allow easy adjustment of the medication list template. The second was partial disagreement with the recommendation (see hypothesis 9).

That’s a problem of the system, we cannot simply change this (…). That’s beyond our possibilities (GP).

#### Hypothesis 7: the information material for patients increased the self-management abilities of patients and reduced language barriers and difficulties of comprehension

We developed various materials to sensitize patients for medication-related issues in order to increase patients’ self-management abilities and to address language barriers and difficulties of comprehension. A multilingual info-tool on the tablet PC was provided to the practices, focusing on the importance of a comprehensive medication list and safe medication use as well as posters calling upon patients to always have their medication list with them and brown paper bags with an imprint encouraging patients to bring their medication packages to the appointment for SMC.

In the survey, 90% of the GPs agreed that patients’ self-management abilities were a relevant determinant and 76% did so concerning the language barrier. Difficulties of comprehension were not considered a relevant determinant by 43% of GPs. The question whether these determinants had been successfully modified by the provided materials was affirmed by 80% of GPs regarding the self-management abilities, by 70% regarding the language barrier and by 90% regarding the difficulties of comprehension (Table 1, item 6–8).

The interview statements were in line with these survey findings concerning the posters and the brown bag, but contradictory concerning the info-tool on the tablet. While most interviewees found the posters and paper bags useful, they reported many problems with the use of the info-tool on the tablet PC mainly because of problems with the internet connection and the suitability for elderly patients.

I think mainly the posters, that they should always have their medication list with them, maybe this sensitised them [the patients]. That was good! (MA)

Almost none of our patients was able to use this tablet themselves. I think the medical assistant did it with them and read it to them or showed it to them (GP)

The analysis of the log file showed logins for 49 different patients (34.3% of the patients of the intervention group). The tool was used 11 times in Turkish. The average login time was 5.9 (0.5–34.7) minutes.

#### Hypothesis 8: implementation of the recommendations has improved

When asking the participants what effect the study had on the processes in their practice, they frequently answered that it had increased their awareness and that they adhered to the recommendations more consistently than before.

It’s not that we have not done this before. We have always done this in patients with dementia or when other problems played a role and when we believed that something is going wrong. But I think we will do it more often in the future (GP).

Of course it is our job to take care that the medications fit and so on. But due to the study I was sensitised to do this more often. And in that respect it helped me (GP).

Some MAs also reported that the study had led to a change of the roles and tasks of the practice staff which is also a hint that implementation of the recommendation has increased in the practice.

We ask more about it and are more interested in it. Before it was only the problem of the doctor and now we do it as well (MA)

Some reported to combine the recommendations now regularly with already established treatment programmes such as disease management programmes or yearly check-ups.

We made it a rule – based on the experience – to let [patients] bring their medications for the DMPs (…). Because you saw how necessary it is and, yes, how many dangerous things happen as well, I think (GP).

The risk patients come any way, at least once per year for the check-up (…) and I think you can combine this very well (…). The only thing is that we, the assistants, have to accustom ourselves to telling the patients, when they make an appointment: Bring your medications with you! (MA)

I think we will adopt this to 100%, mainly the thing with the check-ups, when patients come anyway (MA).

Another positive issue which was mentioned repeatedly was that the patients’ use of their medication list had improved.

The patients made more efforts to understand and update their medication list. This did a lot for us (…), that was surprisingly positive. I wouldn’t have thought this, to be honest. (GP)

I think the patients take it more serious now, that they carry the medication list with them (MA)

I think many became aware what it means to have a medication list and to show it to everyone (GP).

#### Hypothesis 9: the implementation of the recommendations leads to improved health outcomes

We did not examine health outcomes such as hospitalization or mortality in the trial but assumed that the implementation of evidence-based recommendations will lead to improved health outcomes. Yet, an indication of the usefulness of the recommendations can be derived from the available data. The GPs (*n* = 8) documented in total 116 appointments for SMC, 99 brown bag reviews and 107 medication reviews (Table 2). According to this documentation, about half of the patients required information and in 43% of the cases, the GPs received useful information during the appointment for structured medication counselling. Irregularities during the brown bag review and a change of the medication were documented for 20% of the cases.

**Table 2:** Documentation of structured medication counselling and medication reviews

	Yes % (*n*)*	No % (*n*)*
1. Did you conduct structured medication counselling with this patient?	96.7 (116)	3.3 (4)
1.1 If yes, did the patient require any information?	53.4 (62)	46.5 (54)
1.2 If yes, did you receive any useful information from the patient or his/her relatives?	43.1 (50)	56.9 (66)
2. Did you conduct a complete inventory of the medication actually taken by the patient (“brown bag review”)?	82.5 (99)	17.5 (21)
2.1 If yes, did the patient bring his medication packages to the practice for this purpose?	74.7 (74)	25.3 (25)
2.2 If yes, were there any irregularities?	20.2 (20)	29.8 (79)
2.3 If yes, did you receive useful information due to the “brown bag review”?	31.3 (31)	67.7 (67)
2.4 If yes, did the brown bag review result in useful instructions for the patient?	43.4 (43)	56.6 (56)
3. Did you give the patient an updated medication list at the end of the appointment?	84.2 (101)	15.8 (19)
4. Did you review the medication of the patient systematically?	89.2 (107)	10.8 (13)
4.1 If yes, did you use the checklist for medication reviews provided by the study?	59.8 (64)	40.2 (43)
4.2 Did you use any other instrument for the medication review?	17.8 (19)	82.2 (88)
4.3 Did the medication review result in a change of the medication?	21.5 (23)	78.5 (84
4.4 If yes, what changes did you make?		
Stopping a medication	34.8 (8)	
Prescription of a new drug	30.4 (7)	
Change of the dosis	47.8 (11)	
Change of application	0.0	
Other	13.0 (3)	

*Percentages refer to 120 medication counselling sessions documented by 8 GPs

In the interviews, the respondents mentioned both negative and positive aspects of the recommendations: They expected that medication reviews would help to reduce polypharmacy and to avoid ADR but also found it time consuming and only necessary for specific patients.

They agreed that the medication list is an important instrument for communication mainly among doctors (less for the patients), but some also stated that in their opinion, it was not necessary that patients carry the medication list with them as it was saved in the practice computer and many preferred less or different information on the medication list than the minimum standards defined by the template to keep it “simple and clear”.

Concerning structured medication counselling, they appreciated the gain of information, the improvement of the doctor-patient relationship, the sensitization of the patients and the saving in time due to the structured process. Yet, they also thought that a separate appointment for structured medication counselling was not necessary as the targeted patients already visit the practice frequently and talking about medication is already something they do, even if not in such a structured way. Some expressed fears to unsettle patients by giving too much information.

Many patients are unsettled, even by the package insert (…) The patients are not able to understand all this (…) you weigh up, how is the benefit and then we make the decision. But I take the decision and we don’t know if there will be a side effect, but they have to trust me that the medication is right for them. They are not able to understand all this, I don’t even know if they understand me. If I would list all side effects (…) they would be very concerned (GP).

One interviewee expressed doubts about the evidence and the effect of the recommendations.

This is my next question: What’s the point of it? What do you want to reach? That less patients die from side effects or what? (…) Of course we are interested in treating our patients better, but to talk about medication just like this? (…). Is there any statistical prove that more patients become sick from drug administration? Is this certain? I can’t judge this. (GP).

## Discussion

Systematic reviews on interventions to improve polypharmacy frequently come to the conclusion that the effects of the interventions are variable and conflicting [14, 33–36]. In the logic of “tailoring”, the effectiveness of implementation strategies will increase if the barriers for adopting the desired behaviour are overcome or facilitating factors are used appropriately. This process evaluation aimed to explain the low effectiveness of a tailored programme. From the results, several suggestions for modification of the TP can be derived which we describe below in accordance with the “framework and coding system for modifications and adaptations of evidence-based interventions” published by Stirman et al. It distinguishes the following key questions: *By whom* is *what* (*content*, *context or evaluation*) at what *level of delivery* modified and what is the *nature of content modifications* [17]?

### By whom are modifications made?

The modifications reported in this article are suggestions of the researcher who designed and evaluated the TP.

### Modifications of context and level of delivery

Bringing medication lists in line with minimum standards failed among others, because the necessary software functions were not provided due to limited resources of the trial. In Germany, this barrier is currently being addressed by a nation-wide project which aims at implementing a standardized medication list in Germany [37]. The perspective of this change in the health care system might have influenced the target group’s motivation of making changes themselves and should have been respected by the TP.

Concerning the quality of the written reports on the implementation action plans, it became apparent that more guidance for this process is needed. This could be realized by conducting outreach visits instead of a workshop. Outreach visits could reduce the efforts for participation in the training and guidance for the analysis of the individual barriers and the elaboration of the implementation action plans could be offered e.g. by defining standard operating procedures for medication management in primary care practices.

### Modification of content by integrating the intervention into another approach

There is evidence that outreach visits are an effective educational strategy [38]; however, large-scale implementation of outreach visits is resource intensive but could be integrated in quality management systems, which are obligatory in Germany, such as the “European practice assessment” [39].

### Modification of content by lengthening and extending elements

Targeting patients who profit most from intensified care was another aspect of the intervention which ought to be improved. We tried to support this process by analyzing insurance claim data and by the individual assessment of the patients by the GPs. Yet, time-consuming procedures such as the brown bag review and systematic medication reviews resulted in only 20% of the cases in an actual change of the medication; a benefit concerning information exchange was perceived in 31–43% of the cases. The barrier of defining the target group has been identified in similar studies [40], so future research should focus on the development of methods for targeting or tailoring interventions on a more individual level.

### Modification of content by adding elements

The determinants we intended to modify are similar to those targeted in comparable studies, such as routine, knowledge or professional communication [6, 41], and also the strategies used in other TPs focusing on polypharmacy, e.g. academic detailing, education, treatment algorithms, patient information leaflets and paper bags, are similar to the strategies we selected [42, 43]. However, we did not involve pharmacists, a frequently used strategy to improve prescribing with variable effects [13], and did not address barriers related to inter-professional and inter-sectoral collaboration. These barriers are certainly of relevance in multimorbid patients with polypharmacy, but difficult to modify with the resources we had in our project. It would be desirable that future research projects with adequate funds focus on such aspects.

### Modification of content by loosening structure

The interviews showed that not all participants were completely convinced about the purpose of the evidence-based recommendations which might be related to insufficient targeting, because an added benefit of adhering to the recommendations was only evident for particular patients. But also the nature of the recommendations, which all comprised a certain standardization and structuring of care processes, may have provoked resistance, because principles of evidence-based medicine (EBM) are sometimes difficult to apply to highly complex patients. While EBM “tends to depict the patient’s illness as a fixed entity with more or less stables properties” [44] and as a consequence tries to define standard treatments for standard situations, several statements indicate that the interviewees were reluctant to standardizing care in this patient group, e.g. to using checklists or to structuring consultations—a view that is supported by parts of the scientific community. For instance, Greenhalgh et al. point out that “evidence-based discussions about options for tests and treatments rarely take full account of which people and perspective the patient would like to bring into the discussion” [44]. From our perspective, this view points out the misunderstanding that standardizing care processes are equivalent with standardizing the content of care. Clinical experience and patient preferences are crucial in an EBM approach. The recommendations we chose make suggestions on process level to ensure that there is room for communication, documentation and critical review of medications and that treatment decision is based on a thorough analysis of the individual situation, needs and preferences of the patient. They do not imply any specific treatment recommendations, just as “shared decision making” does not mean that every patient under any circumstances has to be involved into decision making. It is well known that some patients prefer to hand over responsibilities in a rather patriarchic patient-physician relationship. In such a case, “shared decision making” can result into the decision not to provide further information. As a consequence, a critical discussion with the target group about the purpose of evidence-based recommendations and the intention behind structuring care processes should be part of implementation processes.

### Modification of content by tailoring

The educational material for patients was generally considered useful but could have been improved by offering different formats for different target groups, e.g. pamphlets additionally to the electronical info-tool or different sizes of the posters. There is evidence that tailored educational material is in some cases superior to non-tailored formats [45].

### Modification of evaluation

**Fig. 2:**
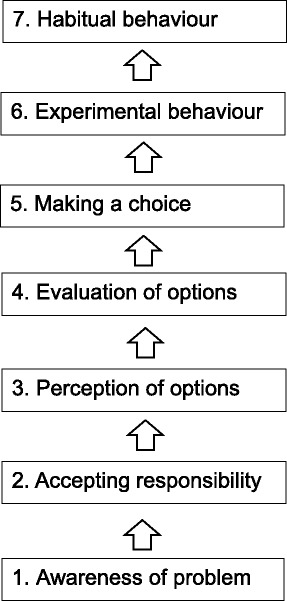
Behaviour change model according to [46]

The raise of awareness was the most frequently mentioned effect of the TP. Several theories state that “becoming aware” is the first necessary step of behaviour change (compare Fig. 2). Although we tried to induce the subsequent steps of behaviour change by the elaboration of the implementation action plans, it is possible that we did not observe a measurable improvement of implementation because the follow-up time was too short and more time for behaviour change was needed. On the other hand, some interviewees reported to have integrated structured medication counselling into existing routine procedures, so they reported “habitual behaviour”. This is in line with the analysis of the primary outcome which showed a positive effect only for medication counselling [28].

### Limitations

Some limitations of this study have to be taken into account: Comparing the findings from the different data sources we used, it was striking that 70–90% of the GPs affirmed the usefulness of all strategies, even when determinants were considered not relevant by the majority of the respondents. Thus, the informative value of the survey is limited, also due to the small number of GPs completing the survey. In the interviews, heterogeneous views on the strategies and determinants were found. Some determinants were perceived as irrelevant and some strategies as not useful by a considerable number of health care professionals leading to the questions whether the methods used to identify determinants and strategies were appropriate and effective. For example, it is possible that involving the target group into the final selection of determinants and strategies could have increased the perceived relevance and acceptance. This aspect has been examined in an international process evaluation combining the findings from the five TICD trials [43].

## Conclusions

While most of the hypotheses specified by the logic model of the TP were confirmed by the survey, the combination of qualitative and quantitative methods resulted in a more detailed understanding of the target group’s view on the TP and the recommendations. It seemed that the TP had successfully induced the first step of behaviour change which is raising awareness while habitual behaviour was reported for only one of the recommendations. Several suggestions for modification of the TP could be deduced, such as conducting outreach visits instead of a workshop, improved targeting, provision of evidence, integration of tools into the practice software and the creation of information materials in tailored formats.

## Additional files

Additional file 1: Checklist for Structured Medication Counselling. (ZIP 528 kb)
Additional file 2: Checklist for Medication Review. (ZIP 247 kb)

## Abbreviations

ADR: Adverse drug reactions
EBM: Evidence-based medicine
GP: General practitioner
MA: Medical assistant
MAI: Medication appropriateness index
SMC: Structured medication counselling
TICD: “Tailoring Interventions for Chronic Diseases” project
TP: Tailored programme
