# Supplementary material for: A tailored programme to implement recommendations for multimorbid patients with polypharmacy in primary care practices—process evaluation of a cluster randomized trial
Source: Implement Sci. 2017 Mar 6;12:31. doi: 10.1186/s13012-017-0559-y (PMC5339959; doi:10.1186/s13012-017-0559-y)
Supplement: Additional file 1: — Checklist for Structured Medication Counselling. (ZIP 528 kb) [file 13012_2017_559_MOESM1_ESM.zip › additional file 1_checklist SMC_English translationR1.pdf]

Checklist for Structured Medication Counselling

„Brown Bag Review“		
**Preparation: When making an appointment for medication counselling with the patient do not forget …**	**Done?** Yes	No
… to remind the patient to bring all medication packages he/she is using to the appointment (especially over-the-counter drugs)		
… to remind the patient to bring the medication list he/she is using		
… to involve relatives if necessary		
**Conduction** Please check for each package …	**Pecularities?** Yes	No
… if the medication packages the patient brought are consistent with the medication list (are their additional or lacking drugs?)		
… how often the patient takes the drug (daily, if needed, does he or she forget to take it etc.)		
… which dosage the patient takes		
… at what time of the day the patient takes the medication		
… if the patient has problems administering the medication (e.g. splitting tablets, administering drops, using inhalators, injecting insulin etc. If necessary let the patient show you how he/she uses the medication)		
… if the order duration is plausible		
… if the „best-buy“ date is exceeded. If yes, discard the package and point it out to the patient)		
**Medical Consultation** Clarify during the consultation if …		
**Effects**	**Yes**	**No**
… whether the medication has the desired effects		
… whether undesired effects of the medications have occured		
**Need for support**	**Yes**	**No**
… the patient needs support with the administration of the medications (especially if there were pecularities during the brown bag review)		
**Need for information**	**Yes**	**No**
… the patient knows for what reason he/she takes each medicament		
… the patient knows what he/she has to do if he/she has forgotten to take a dosage or if he/she has taken too much		
… the patients would like to have more information about his/her medication (e.g. about possible adverse reactions or how the medication generates its effects)		
**Beliefs about medication**	**Yes**	**No**
… the patients has concerns about his medication.		
… has the feeling that the medication is useful for him / her.		
**Updating the medcation list**		
**At the end of the appointment: Did you**	**Yes**	**No**
… hand out an updated medication list to the patient?		
… update the medication list stored in the practice?		
… instruct the patients how to use the medication list (e.g. to take it always along, to discard old medication lists, to add medications he / she buys without prescriptions or receives from another doctor) ?		
