# Supplementary material for: A tailored programme to implement recommendations for multimorbid patients with polypharmacy in primary care practices—process evaluation of a cluster randomized trial
Source: Implement Sci. 2017 Mar 6;12:31. doi: 10.1186/s13012-017-0559-y (PMC5339959; doi:10.1186/s13012-017-0559-y)
Supplement: Additional file 1: — Checklist for Structured Medication Counselling. (ZIP 528 kb) [file 13012_2017_559_MOESM1_ESM.zip › additional file 1_checklist SMC_German originalR1.pdf]

Checkliste für strukturierte Arzneimittelgespräche

**Bestandsaufnahme (Brown Bag Review)**		
**Vorbereitung:**<br>Wenn Sie den Patienten einbestellen, denken Sie daran …	**Erledigt?**<br>Ja	 Nein
… den Patient an die Mitnahme der Medikamente zu erinnern (v.a. an frei verkäufliche Medikamente).	☐	☐
… den Patient an die Mitnahme des Medikamentenplans zu erinnern.	☐	☐
… wenn nötig Angehörige mit einzubinden.	☐	☐
**Durchführung**<br>Überprüfen Sie für jede Medikamentenpackung …	**Auffälligkeiten?**<br>Ja	 Nein
… ob die mitgebrachten Medikamente mit dem Medikamentenplan übereinstimmen (gibt es zusätzliche oder fehlende Medikamente?)	☐	☐
… wie oft der Patient das Medikament einnimmt.<br>(täglich, nach Bedarf, werden Einnahmen vergessen etc.)	☐	☐
… in welcher Dosierung der Patient das Medikament einnimmt.	☐	☐
… zu welchen Tageszeiten der Patient das Medikament einnimmt.	☐	☐
… ob der Patient Probleme bei der Einnahme des Medikaments hat.<br>(Tabletten teilen, Tropfen einnehmen, Inhalator verwenden, Insulin spritzen etc.). Lassen Sie sich ggf. die Anwendung zeigen.	☐	☐
… ob die Verordnungsdauer plausibel ist.	☐	☐
… ob das Mindesthaltbarkeitsdatum überschritten ist. Verwerfen Sie ggf. abgelaufene Medikamente und weisen Sie den Patienten darauf hin.	☐	☐
**Arztgespräch**<br>Klären Sie im Gespräch, ob …		
**Effektivität**	**Ja**	**Nein**
… die Medikamente die gewünschte Wirkung erzielen.	☐	☐
… unerwünschten Wirkungen der Medikamente aufgetreten sind.	☐	☐
**Unterstützungsbedarf**	**Ja**	**Nein**
… der Patient Unterstützung bei der Einnahme der Medikamente benötigt, v.a. bei Auffälligkeiten bei der Bestandsaufnahme.	☐	☐
**Informationsbedarf**	**Ja**	**Nein**
… weiß, wofür er seine Medikamente einnimmt.	☐	☐
… was zu tun ist, falls er vergisst, das Medikament einzunehmen oder zu viel einnimmt.	☐	☐
… mehr Informationen zu seinen Medikamenten haben möchte (z.B. über unerwünschte Wirkungen oder Wirkungsweise).	☐	☐
**Einstellung zur Medikation**	**Ja**	**Nein**
… Bedenken bei der Einnahme seiner Medikamente hat.	☐	☐
… das Gefühl hat, dass die Medikamente helfen.	☐	☐
**Aktualisierung des Medikamentenplans**		
**Wurde am Ende des Termins …**	**Ja**	**Nein**
… dem Patient ein aktueller Medikamentenplan ausgehändigt?	☐	☐
… der Medikamentenplan der Praxis aktualisiert?	☐	☐
… der Patient im Umgang mit dem Medikamentenplan angeleitet?<br>(z.B. den Plan stets mit sich zu führen, alte Pläne zu verwerfen, Selbstmedikation oder Verordnungen durch andere Ärzte zu ergänzen)	☐	☐
