# Supplementary material for: A tailored programme to implement recommendations for multimorbid patients with polypharmacy in primary care practices—process evaluation of a cluster randomized trial
Source: Implement Sci. 2017 Mar 6;12:31. doi: 10.1186/s13012-017-0559-y (PMC5339959; doi:10.1186/s13012-017-0559-y)
Supplement: Additional file 2: — Checklist for Medication Review. (ZIP 247 kb) [file 13012_2017_559_MOESM2_ESM.zip › additional file 2_checklist medication review_English translationR1.pdf]

Checklist for medication review

Please check for each drug of the patient:

Item / Drug	1	2	3	4	5	6	7	8	9	10	11	12	Notes
Indication<br>Is there an indication for the drug?													
Effectiveness<br>Is the drug effective for the indication?													
Dosage<br>Is the dosage correct (kidney function!) ?													
Application<br>Is the application correct and feasible?													
Interactions<br>Are there relevant interactions with other drugs?													
Co-morbidities<br>Are there interactions or contraindications due to co-morbidities?													
Double prescriptions<br>Is the substance listed several times?													
PRISCUS List<br>Is the drug listed on the PRISCUS List?													
QT-Drugs<br>May the drug cause QT prolongation?													
Sedation<br>Does the drug have sedative effects?													
Anticholinergic Drugs<br>Does the drug have anticholinergic effects?													
Under-supply<br>Does the patient have a disease or symptoms which are not sufficiently treated?													

* Modified according to the Medication Appropriateness Index

PRISCUS List (alphabetic order)

Acemetacin	Dimenhydrinat	Maprotilin	Prasugrel
Alprazolam	Dimetinden	Medazepam	Prazepam
Amitryptilin	Diphenhydramin	Meloxicam	Prazosin
Baclofen	Doxazosin	Methyldopa	Reserpin
Bromazepam	Doxepin	Naftidrofuryl	Solifenacin
Brotizolam	Doxylamin	Nicergolin	Sotalol
Chinidin	Ergotamin	Nifedipin	Temazepam
Chloralhydrat	Etoricoxib	Nitrazepam	Terazosin
Chlordiazepoxid	Flecainid	Nitrofurantoin	Tetrazepam
Chlorphenamin	Flunitrazepam	Olanzapin	Thioridazin
Clemastin	Fluoxetin	Oxazepam	Ticlopidin
Clobazepam	Fluphenazin	Oxybutynin	Tolterodin
Clomipramin	Flurazepam	Paraffin	Trancylpromin
Clonidin	Haloperidol	Pentoxifyllin	Triazolam
Clozapin	Hydroxyzin	Perphenazin	Trimipramin
Diazepam	Imipramin	Pethidin	Triprolidin
Digoxin	Indometacin	Phenobarbital	Zaleplon
Dihydroergocryptin	Ketoprofen	Phenylbutazon	Zolpidem
Dihydroergotoxin	Levomeprazin	Piracetam	Zopiclon
Dikaliumclorazepat	Lorazepam	Piroxicam	
