# Supplementary material for: A tailored programme to implement recommendations for multimorbid patients with polypharmacy in primary care practices—process evaluation of a cluster randomized trial
Source: Implement Sci. 2017 Mar 6;12:31. doi: 10.1186/s13012-017-0559-y (PMC5339959; doi:10.1186/s13012-017-0559-y)
Supplement: Additional file 2: — Checklist for Medication Review. (ZIP 247 kb) [file 13012_2017_559_MOESM2_ESM.zip › additional file 2_checklist medication review_German originalR1.pdf]

Checkliste für Medikationsprüfungen

Bitte prüfen Sie für jedes Medikament des Patienten:

**Medikament**<br>**Item**	**1**	**2**	**3**	**4**	**5**	**6**	**7**	**8**	**9**	**10**	**11**	**12**	**Medikation gesamt / Notizen**
Indikation<br>Gibt es für das Medikament eine Indikation?													
Effektivität<br>Ist das Medikament hinsichtlich der Indikation effektiv?													
Dosierung<br>Ist die Dosierung für korrekt (Nierenfunktion!)?													
Applikation<br>Ist die Applikation korrekt und praktikabel?													
Wechselwirkungen<br>Gibt es Wechselwirkungen mit anderen Medikamenten?													
Begleiterkrankungen<br>Gibt es Wechselwirkungen / Kontraindikationen durch Begleiterkrankungen?													
Doppelverordnungen<br>Ist der Wirkstoff mehrfach verordnet?													
Priscus – Liste<br>Steht das Medikament auf der Priscus-Liste?													
QT-Zeit<br>Kann das Medikament eine Verlängerung der QT-Zeit bewirken?													
Sedierung<br>Hat das Medikament sedierende Effekte?													
Anticholinergika<br>Hat das Medikament anticholinerge Effekte?													
Unterversorgung<br>Hat der Patient eine Erkrankung oder Beschwerden, die nicht ausreichend medikamentös therapiert sind?													

* Modifiziert nach dem Medication Appropriateness Index

PRISCUS – Liste alphabetisch

Acemetacin	Dimenhydrinat	Maprotilin	Prasugrel
Alprazolam	Dimetinden	Medazepam	Prazepam
Amitryptilin	Diphenhydramin	Meloxicam	Prazosin
Baclofen	Doxazosin	Methyldopa	Reserpin
Bromazepam	Doxepin	Naftidrofuryl	Solifenacin
Brotizolam	Doxylamin	Nicergolin	Sotalol
Chinidin	Ergotamin	Nifedipin	Temazepam
Chloralhydrat	Etoricoxib	Nitrazepam	Terazosin
Chlordiazepoxid	Flecainid	Nitrofurantoin	Tetrazepam
Chlorphenamin	Flunitrazepam	Olanzapin	Thioridazin
Clemastin	Fluoxetin	Oxazepam	Ticlopidin
Clobazepam	Fluphenazin	Oxybutynin	Tolterodin
Clomipramin	Flurazepam	Paraffin	Trancylpromin
Clonidin	Haloperidol	Pentoxifyllin	Triazolam
Clozapin	Hydroxyzin	Perphenazin	Trimipramin
Diazepam	Imipramin	Pethidin	Triprolidin
Digoxin	Indometacin	Phenobarbital	Zaleplon
Dihydroergocryptin	Ketoprofen	Phenylbutazon	Zolpidem
Dihydroergotoxin	Levomeprazin	Piracetam	Zopiclon
Dikaliumclorazepat	Lorazepam	Piroxicam	
